# Supplementary material for: Rare Coding Variants in Patients with Non-Syndromic Vestibular Dysfunction
Source: Genes (Basel). 2023 Mar 30;14(4):831. doi: 10.3390/genes14040831 (PMC10137884; doi:10.3390/genes14040831)
Supplement: Supplementary file 1 [file genes-14-00831-s001.zip › genes-2300342-suppl-new/genes-2300342-Supplementary Information-new.pdf]

Table S1. Adolescent patients with lateral semicircular canal asymmetry and idiopathic scoliosis (Carry et al. 2020).

ID	Age (years)	Curve type	Size of curve (Cobb angle)
1	14.5	Thoracic	47
2	11.6	Thoracic+Lumbar	30/28
3	11.9	Thoracic+Lumbar	43/38
4	14	Thoracic+Lumbar	59/52
5	12	Thoracic+Lumbar	56/37
6	15	Thoracic+Lumbar	41/47
7	17	Thoracic+Lumbar	35/28
8	16	Thoracic+Lumbar	37/14
9	16	Thoracic+Lumbar	42/21
10	16	Thoracic+Lumbar	50/50

Table S4. *OTOP1* (NM_177998.1) exon 6 variants on hg19 chromosome 4 that were identified in US adolescents with asymmetry of the lateral semicircular canals by MRI (n=11).

hg19 position	Reference allele	Alternate allele	cDNA variant	Amino acid variant	Highest MAF	Damaging prediction (dbNSFP42a)	Scaled CADD score
4228586	G	A	c.6C>T	p.(=)	(AU)Afr: 0.035	na	1.26
4228563	G	C	c.29C>G	p.(Ser10Trp)	Bravo: 7.96E-6	SI	14.98
4228547	T	C	c.45A>G	p.(=)	0	na	2.05
4228538	C	T	c.54G>A	p.(=)	(AU)Lat: 3.3E-5	na	3.39
4228526	C	T	c.66G>A	p.(=)	0	na	0.74
4228517	T	G	c.75A>C	p.(=)	0	na	2.21
4228507	A	G	c.85T>C	p.(Ser29Pro)	0	.	4.35
4228493	G	C	c.99C>G	p.(=)	OTH: 0.003	na	1.98
4228456	G	T	c.136C>A	p.(=)	SAS: 0.27	na	4.56

Abbreviations: Afr: African; (AU): All of Us database; CADD: Combined Annotation Dependent Depletion; Lat: Latin American; OTH: gnomAD Other; SAS: gnomAD South Asian; SI, SIFT deleterious. All 11 adolescents were heterozygous for 9 out of 11 variants (except c.45A>G and c.75A>C), suggesting that these variants are in strong linkage disequilibrium. Out of 11 individuals with lateral semicircular canal asymmetry, 10 were diagnosed with idiopathic scoliosis, while one was labeled as a control individual with normal spine.

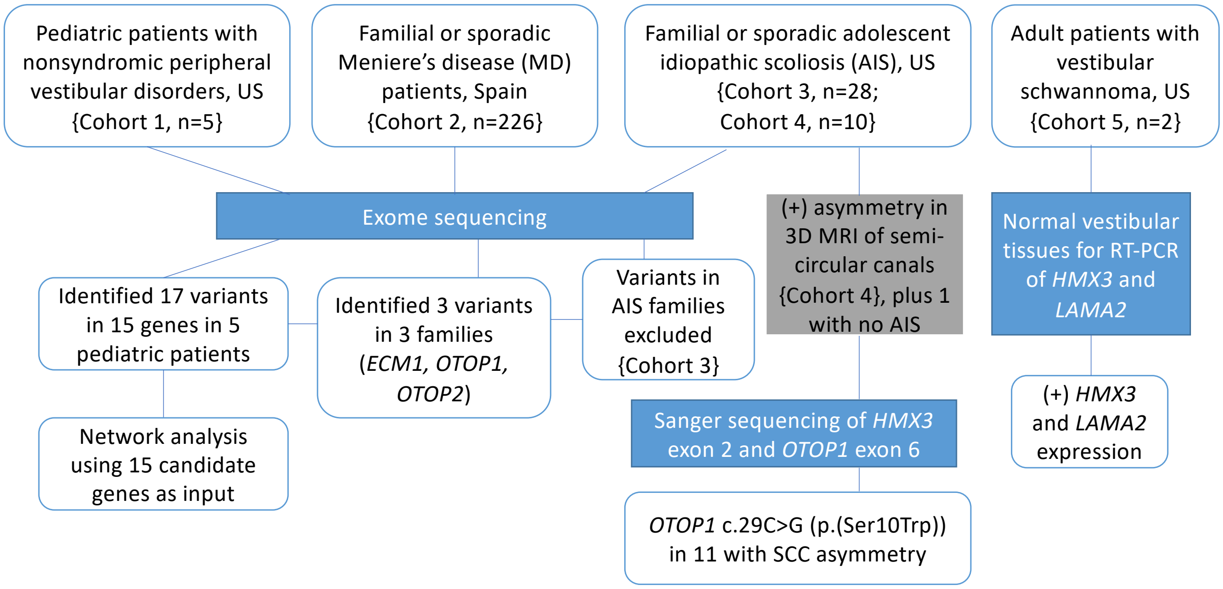

Figure S1. Study flowchart.

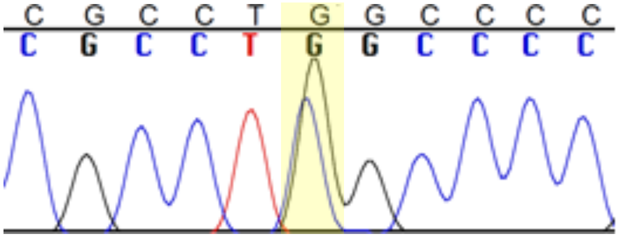

Figure S2. *OTOP1* c.29C>G (p.(Ser10Trp)) variant identified by Sanger sequencing in 11 patients with lateral semicircular canal asymmetry, 10 of whom had idiopathic scoliosis.
